# Correlations between autoantibodies and the ATR-FTIR spectra of sera from rheumatoid arthritis patients

**DOI:** 10.1038/s41598-021-96848-w

**Published:** 2021-09-09

**Authors:** Katarzyna Durlik-Popińska, Paulina Żarnowiec, Iwona Konieczna-Kwinkowska, Łukasz Lechowicz, Józef Gawęda, Wiesław Kaca

**Affiliations:** 1 Department of Microbiology and Parasitology, Institute of Biology, Jan Kochanowski University in Kielce, Kielce, Poland; 2 Rheumatology Clinic ARTIMED, Kielce, Poland; 3 Świętokrzyskie State Provincial Sanitary Inspection, Kielce, Poland

**Keywords:** Diagnostic markers, Predictive markers

## Abstract

Rheumatoid arthritis (RA) is one of the most common autoimmune diseases worldwide. Due to high heterogeneity in disease manifestation, accurate and fast diagnosis of RA is difficult. This study analyzed the potential relationship between the infrared (IR) spectra obtained by attenuated total reflectance Fourier transform infrared spectroscopy (ATR-FTIR) and the presence of autoantibodies and antibodies against urease in sera. Additionally, the wave number of the IR spectrum that enabled the best differentiation between patients and healthy blood donors was investigated. Using a mathematical model involving principal component analysis and discriminant analysis, it was shown that the presence of anti-citrullinated protein antibody, rheumatoid factor, anti-neutrophil cytoplasmic antibodies, and anti-nuclear antibodies correlated significantly with the wave numbers in the IR spectra of the tested sera. The most interesting findings derived from determination of the best predictors for distinguishing RA. Characteristic features included an increased reaction with urease mimicking peptides and a correspondence with particular nucleic acid bands. Taken together, the results demonstrated the potential application of ATR-FTIR in the study of RA and identified potential novel markers of the disease.

## Introduction

Rheumatoid arthritis (RA) is one of the most common immune-mediated rheumatic diseases affecting 0.5–1% of the adult population worldwide (20–50 cases per 100,000 annually). Women have a higher risk of developing RA after 40 years of age and the ratio of prevalence between females and males is approximately^1–3^ 3:1 or 2:1. Clinical manifestations of RA consist of both systemic and joint dysfunctions. Chronic inflammation is due to uncontrolled infiltration by inflammatory and immune cells, and the release of pro-inflammatory mediators such as tumor necrosis factor-α (TNF-α), interleukin-1β (IL-1β), and interleukin-6 (IL-6), which leads to disruption of homeostasis and immune system disorders. In addition, the overexpression of inflammatory cytokines, upregulation of matrix-degrading enzymes (e.g., matrix metalloproteinases and cathepsin), as well as the production of autoantibodies, are thought to play an important role in the hyperplasia of cells in synovial membranes, leading to progressive tendon, cartilage, and bone tissue damage, mostly in the small joints of the hands and feet. The etiology of RA is multifactorial, with a significant contribution from genetic and environmental factors that result in complex disease pathology and heterogenicity of the symptoms^4,5^. Without appropriate treatment, RA may lead to long-term joint damage, chronic pain, heart problems, disability, and excess mortality^6,7^. Due to the high heterogeneity of RA disease, precise diagnosis is difficult and time-consuming^8^. An early and accurate diagnosis of RA is essential for appropriate treatment and symptomatic relief^9^. Currently, the diagnosis of RA is based on an interview with the patient, imaging tests (X-ray, ultrasound, and magnetic resonance imaging), and tests for the detection of clinical inflammatory biomarkers that determine the general inflammatory state of RA, and the identification of RA autoantibodies^7^. One of the most well-known autoantibodies in RA is rheumatoid factor (RF), and it is a marker included in the major classification criteria: 1987 American College of Rheumatology (ACR) and 2010 ACR/European League Against Rheumatism (EULAR). RF is defined as an immunoglobulin against the Fc region of the IgG class of antibodies, and is routinely screened in diagnostics laboratories to differentiate RA from other diseases with similar symptoms^6^. However, despite RF owing its name to being first detected in RA patients, it has also been found in patients with other rheumatic diseases such as Sjogren’s syndrome and systemic sclerosis, as well as in non-rheumatic autoimmune conditions and a variety of infectious diseases^10^. However, RF is not always detected in RA patients, which can make it difficult to diagnose the disease. Studies show that the sensitivity and specificity of RF are approximately 64% and 77%, respectively^11^. RF often co-occurs with anti-citrullinated proteins antibodies (ACPA). ACPA are directed against citrulline residues on proteins or peptides. ACPA are present in approximately 60%–90% of patients with established RA and 50%–60% of early RA cases, and is an important biomarker included in the widely used ACR/EULAR 2010 classification criteria^6^. It is noteworthy that ACPA has been observed for years in asymptomatic individuals and does not necessarily lead to RA development. Additionally, ACPA can be detected in other rheumatic diseases or may not be present in the serum of RA patients. Studies show that the sensitivity and specificity of ACPA are approximately 62% and 90%, respectively, which makes it a more specific RA marker compared with RF^11^. Despite RF and ACPA playing a significant role in the diagnosis of RA, there is still a need for new biomarkers and diagnostic methods for RA, especially in the seronegative subgroup^11^. Other RA-associated antibodies exist, e.g., antibodies to nuclear antigens (ANA), anti-neutrophil cytoplasmic antibodies (ANCA), anti-collagen type II antibodies, and anti-fibronectin antibodies. However, most of these autoantibodies are not specific for RA and they are also found in the sera of patients with other autoimmune diseases^3,12–14^. Previous studies showed that patients with RA possess an increased level of anti-urease (anti-Ure) antibodies, and the sensitivity and specificity of the sera reaction with six bacterial urease mimicking peptides were approximately 96–100% and 94–98%, respectively^15^. Interestingly, the presence of anti-Ure antibodies is not linked with autoreactivity but instead with bacterial infection. Molecular mimicry between *Proteus mirabilis* urease and collagen type XI suggests linkage between bacterial infection and disease development^16^. The sensitivity and specificity of anti-Ure antibodies indicates the high potential of these antibodies as RA markers^15^.

During these times of high technological development and innovation, new research methods are being sought that can provide insight into the development, course, and diagnosis of heterogeneous diseases such as RA and other autoimmune diseases. One of the most easy, rapid, and sensitive laboratory techniques increasingly used in biological applications is Fourier transform infrared spectroscopy (FTIR)^17^. FTIR has been applied as a noninvasive physical–chemical method for the discrimination, classification, and identification of biological materials. The FTIR spectra of cells, tissues, and fluids reflect the vibrational or rotational motions of specific functional groups or bonds in biochemical components such as proteins, carbohydrates, and lipids. Biological samples produce a unique fingerprint-like IR spectra, including broad and superimposed absorbance bands over the entire mid-infrared (mid-IR) spectral region of^18^ 4000–650 cm^1^. Obtained spectra can be divided into groups of components with typical absorption bands in the wave number windows (W): W1 corresponds to fatty acids (wave number range 3000–2800 cm^−1^), W2 corresponds to proteins and peptides (wave number range 1800–1500 cm^−1^), W3 corresponds to proteins, phosphate-carrying compounds, and fatty acids (wave number range 1500–1200 cm^−1^), W4 corresponds to carbohydrates (wave number range 1200–900 cm^−1^), and W5 corresponds to specific peaks unique to the sample (wave number range 900–750 cm^−1^)^19^. In medical sciences, FTIR is mostly employed by researchers to improve the diagnosis and treatment of cancer, but is also utilized for other chronic illness such as autoimmune disorders^20,21^. Earlier studies on the possible applications of FTIR in the diagnosis of RA showed that this technique was able to distinguish RA patients from healthy blood donors^22^.

In the present study, it was shown that sera from RA patients had unique IR spectral patterns that correlated with typical RA autoantibody biomarkers such as ACPA, RF, and less specifically for RA, ANA, and ANCA, that were detected in autoimmune diseases including RA^3^. Moreover, it was shown that the IR spectra of patients’ sera correlated with antibodies against bacterial urease mimicking peptides (anti-Ure), which were previously described as promising tools in RA diagnostics^15^.

## Results

### Presence of autoantibodies in sera samples

To obtain a clear picture of the relationship between autoantibodies and anti-Ure antibodies, and IR spectra, samples of the sera used in this study were analyzed for the presence of autoantibodies: RF, ACPA, ANA, ANCA, and anti-Ure antibodies. Most patient sera were RF positive (74%) with a median titer of 48 IU/mL (< 30–128) and two of the healthy blood donor sera were RF positive. All patient sera samples and five of the healthy blood donor sera (31%) were ACPA positive. The median ACPA concentration in sera was 146.32 U/mL (27.32–908.28) and 9.42 U/mL (7.14–19.44) for patients and healthy blood donors, respectively. ANA and ANCA were less frequently detected. ANA were present in 47% of patients’ sera and one serum sample from a healthy blood donor. ANCA were present in 30% of patients’ sera and in two sera samples from healthy blood donors. Patient sera contained significantly greater levels of anti-Ure antibodies compared with healthy blood donors (*p* < 0.05). The dot blot median reaction values measured by the grayscale were 34.07 (27.63–43.22) and 9.62 (7.68–12.14) for patients and healthy blood donors, respectively. Analysis of the correlation of occurrence of the tested antibodies showed a significant relationship (*p* < 0.05) between RF, ACPA, ANA, and anti-Ure antibodies, where the strongest relationship was observed between RF and ACPA (r = 0.55), followed by anti-Ure and ACPA (r = 0.5), anti-Ure and ANA (r = 0.42), anti-Ure and RF (r = 0.42), and ANA and ACPA (r = 0.41). The weakest relationship was observed between RF and ANA (r = 0.36). There was no correlation between ANCA and other antibodies.

### Differentiation of sera based on autoantibodies and anti-urease antibodies

**Figure 1:**
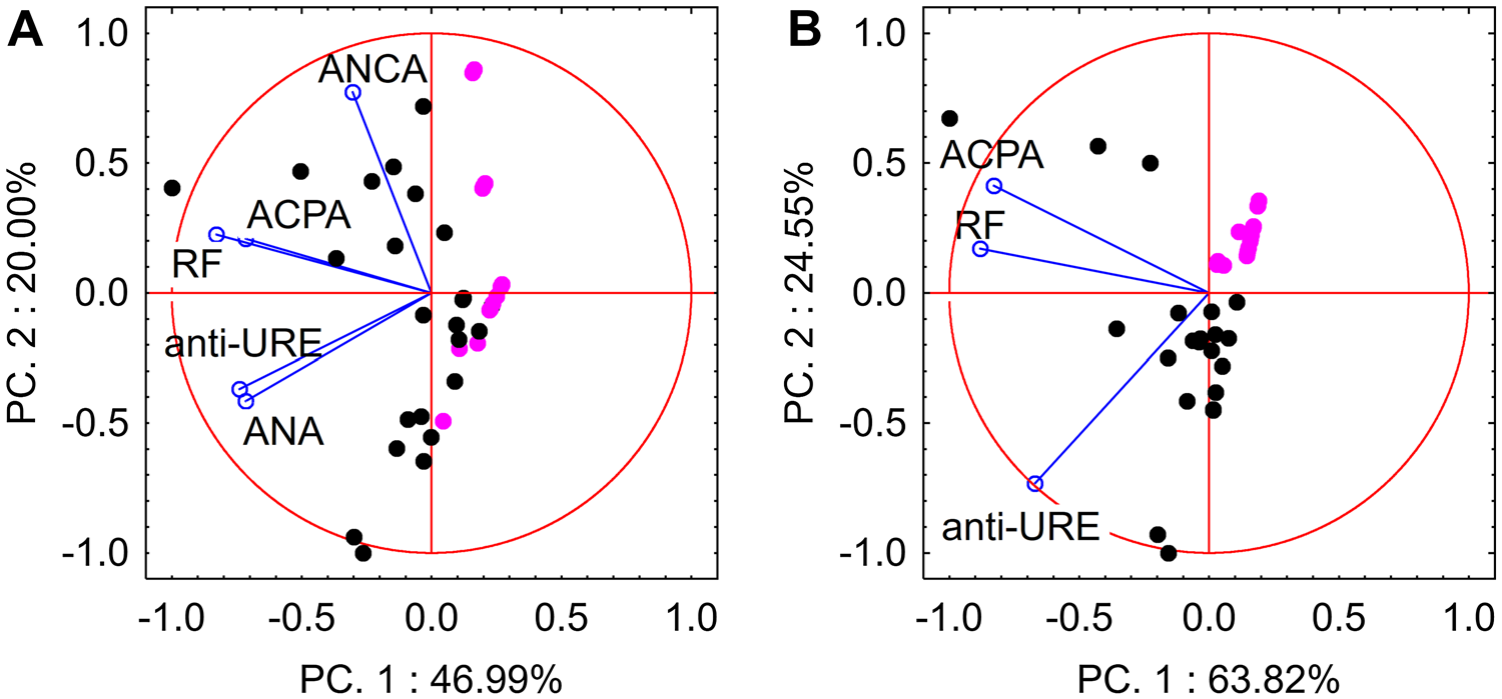
The PCA biplot displays the correlations: (**A**) between the five clinical markers and individual serum samples, PC1 and PC2 accounted for 66.99% of the total variance, (**B**) between the three clinical markers and individual serum samples, PC1 and PC2 accounted for 88.37% of the total variance. Rheumatoid arthritis patients’ sera samples are marked in black (n = 23) and healthy blood donors’ sera samples are marked in pink (n = 16).

Based on information about the presence of autoantibodies and anti-Ure antibodies in sera samples, next, it was investigated which group of antibodies could best separate the sera into two categories: patients and healthy blood donors. The principal component analysis (PCA) loadings and scatter plots displayed five features and individual samples exhibited the first two components (Fig. 1). The first principal component (PC1; 46.99%), the second principal component (PC2; 20%), and the third principal component (PC3; 17.8%) accounted for 84.8% of the total variance of the five features. The loading plot also demonstrated that RF, ACPA, ANA, and anti-Ure antibodies were associated with PC1, while ANCA was associated with PC2. The ANCA and ACPA markers were also related to PC3. In Table 1, the presence and concentration of these five features in the sera of the study group are shown. The first two markers, RF and ACPA, had significantly higher median levels in patients than in healthy blood donors. Additional markers such as ANA and ANCA demonstrated more positive results in RA patients than healthy blood donors. Taken together, the markers did not clearly distinguish the patient group from the blood donor group (Fig. 1a).

**Table 1:** Baseline information of RA patients and healthy blood donors.

	RA patients n = 23	Healthy blood donors n = 16
Male	4	0
Female	19	16
Age (years)	63 ± 13	45 ± 5
25-Hydroxy Vit. D (ng/mL)	24.1 (14.34–37.1)	–
Calcium total	9.46 (9.06–9.68)	–
HDL	56.2 (50.2–76)	–
LDL	107.08 (98.48–135.04)	–
**Therapeutics (% of patients)**		
Methotrexate	68	–
Methylprednisolone	44	–
Tocilizumab	19	–
Denosumab	13	–
Etanercept	6	–
Chloroquine	6	–
Leflunomide	6	–
Salazosulfapyridine	18	–
**Serological disease markers shown as median (IQR) or percent**		
CRP (mg/mL)	4.12 (1.33–11.13)	–
ESR (mm/h)	20 (11–44.5)	–
ACPA(U/mL)	146.32 (27.32–908.28)	9.42 (7.14–19.44)
ACPA positive (> 10 U/mL)	100	31
IgM-RF (≥ 30 IU/mL, latex agglutination test)	74	12
IgM-RF Titer (IU/mL)	48 (< 30–128)	–
ANA positive (%)	47	6
ANCA positive (%)	30	12
Anti- URE antibodies (grayscale)	34.07 (27.63–43.22)	9.62 (7.68–12.14)

For this reason, the analysis was performed using a range of variants of the variables. The best discriminant abilities were obtained for the markers RF, ACPA, and anti-Ure antibodies (Fig. 1b).

A multivariate analysis was performed to select clinical variables to distinguish RA patients from healthy donors. Using a discriminant analysis (DA) model, the anti-Ure antibody, RF, and ACPA levels were included, as they were the only independent parameters that were significantly associated with RA (Supplemental information 1). The discriminant values of the model only used these three parameters. These data could be used to distinguish RA patients from healthy donors based on the fact that the mean values of the three parameters were statistically significant (Wilks' Lambda = 0.36941; F = 19.915; *p* < 0.0000). Among the examined parameters, the greatest contribution to distinguishing RA patients from healthy blood donors was from anti-Ure antibodies (lowest partial Wilks’s lambda value). The calculated classification matrix showed that differentiating patients from healthy blood donors had a sensitivity of 91.3% and a specificity of 93.75%.

### Wave number correlates with markers of rheumatic arthritis.

**Figure 2:**
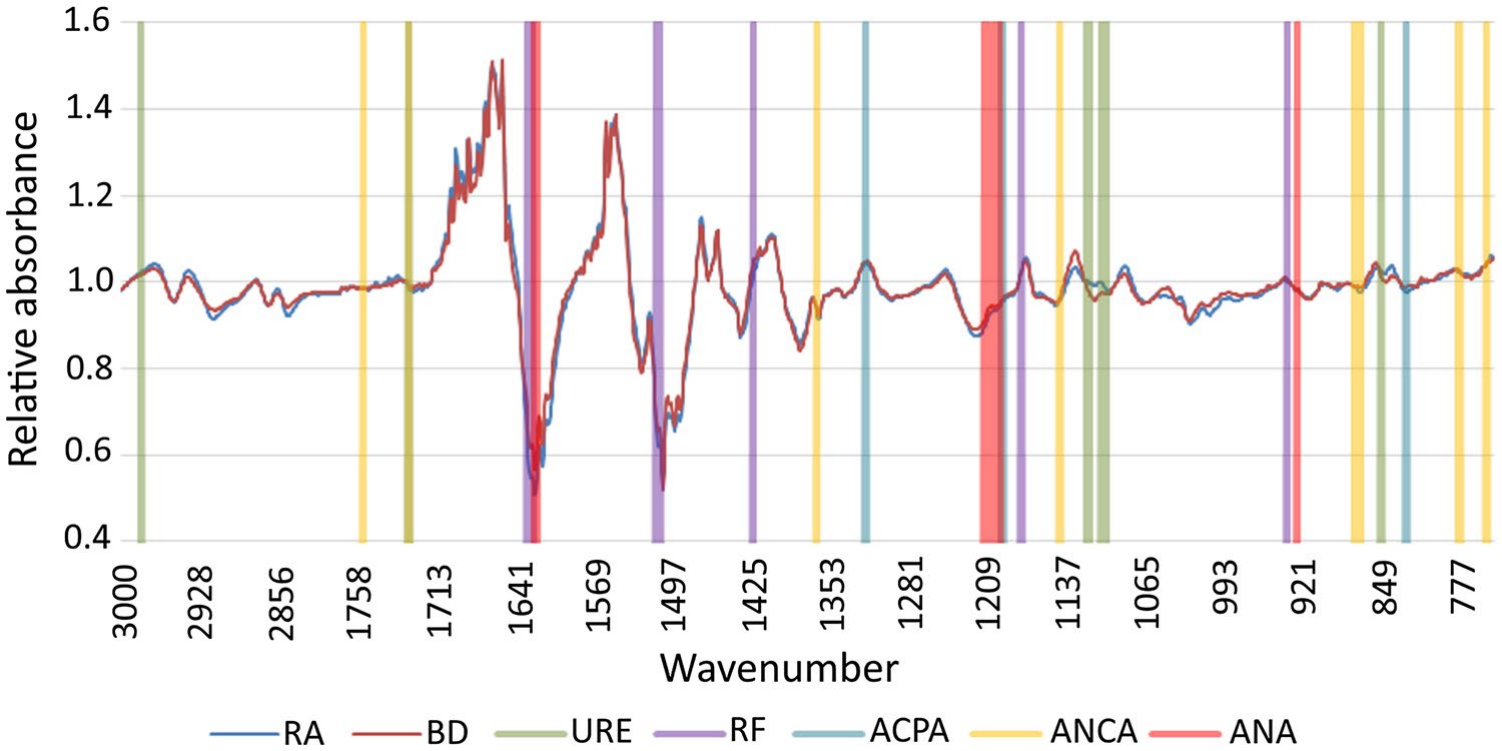
Best predictors characteristic of the examined antibodies, as calculated by the chi2 test.

To determine whether the presence and concentration of the examined antibodies may affect the IR spectra of sera, statistical analyses were performed. Using the chi2 test, the best predictors from the sera spectra were selected to discriminate sera based on the presence and value of these antibodies (Fig. 2).

Next, the obtained predictors were correlated with the corresponding antibodies. Surprising, not all predictors significantly correlated with the corresponding antibodies.

For anti-Ure antibodies, 11 wave number predictors were selected and all showed a positive correlation with the value of these antibodies: 1120 (r = 0.69, *p* < 0.0001), 1105 (r = 0.68, *p* < 0.0001), 1103 (r = 0.67, *p* < 0.0001), 1104 (r = 0.66, *p* < 0.0001), 1106 (r = 0.68, *p* < 0.0001), 1118 (r = 0.66, *p* < 0.0001), 1119 (r = 0.65, *p* < 0.0001), 1107 (r = 0.65, *p* < 0.0001), 852 (r = 0.64, *p* < 0.0001), 2982 (r = 0.68, *p* < 0.0001), and 1682 cm^−1^ (r = 0.62, *p* < 0.0001).

Similarly, for RF, nine characteristic bands were identified. All of the selected predictors were significantly negatively correlated with RF: 1628 (r = *− *0.65, *p* < 0.0001), 1631 (r = *− *0.68, *p* < 0.0001), 1626 (r = *− *0.61, *p* < 0.0001), 1180 (r = *− *0.28, *p* = 0.09), 1511 (r = *− *0.65, *p* < 0.0001), 1510 (r = *− *0.65, *p* < 0.0001), 1627 (r = *− *0.63, *p* < 0.0001), 1425 (r = *− *0.63, *p* < 0.0001), and 938 cm^−1^ (r = *− *0.58, *p* < 0.0001). For ACPA, nine bands were identified and only one did not show a significant correlation with the ACPA concentration : 1341 (r = *− *0.37, *p* = 0.02), 1322 (r = *− *0.46, *p* = 0.003), 1739 (r = *− *0.5, *p* = 0.001), 829 (r = *− *0.71, *p* < 0.0001), 1342 (r = *− *0.4, *p* = 0.01), 1344 (r = *− *0.25, *p* = 0.1), 1343 (r = *− *0.37, *p* = 0.02), 1198 (r = *− *0.56, *p* = 0.0002), and 1197 cm^−1^ (r = *− *0.62, *p* = 0.0001). For ANCA, 10 bands were selected but only half correlated with these autoantibodies: 780 (r = *− *0.32, *p* = 0.005), 1781 (r = *− *0.03, *p* = 0.8), 1739 (r = 0.05, *p* = 0.7), 756 (r = 0.26, *p* = 0.02), 871 (r = *− *0.25, *p* = 0.02), 1367 (r = 0.17, *p* = 0.1), 1147 (r = 0.06, *p* = 0.6), 755 (r = *− *0.3, *p* = 0.007), 781 (r = *− *0.34, *p* = 0.004), and 876 cm^−1^ (r = 0.01, *p* = 0.8); and for ANA, 13 wave numbers were characteristic and 12 of these showed a significant correlation with these autoantibodies: 1213 (r = *− *0.39, *p* = 0.0005), 929 (r = *− *0.17, *p* = 0.1), 1622 (r = *− *0.45, *p* < 0.0001), 1623 (r = *− *0.46, *p* < 0.0001), 1624 (r = *− *0.47, *p* < 0.0001), 1205 (r = *− *0.44, *p* < 0.0001), 1200 (r =  − 0.4, *p* = 0.0004), 1212 (r = *− *0.39, *p* = 0.0005), 1214 (r = *− *0.4, *p* = 0.0003), 1321 (r = *− *0.35, *p* = 0.001), 1201 (r = *− *0.39, *p* = 0.0004), 1204 (r = *− *0.42, *p* = 0.0002), and 1203 cm^−1^ (r = − 0.39, *p* = 0.0004).

**Figure 3:**
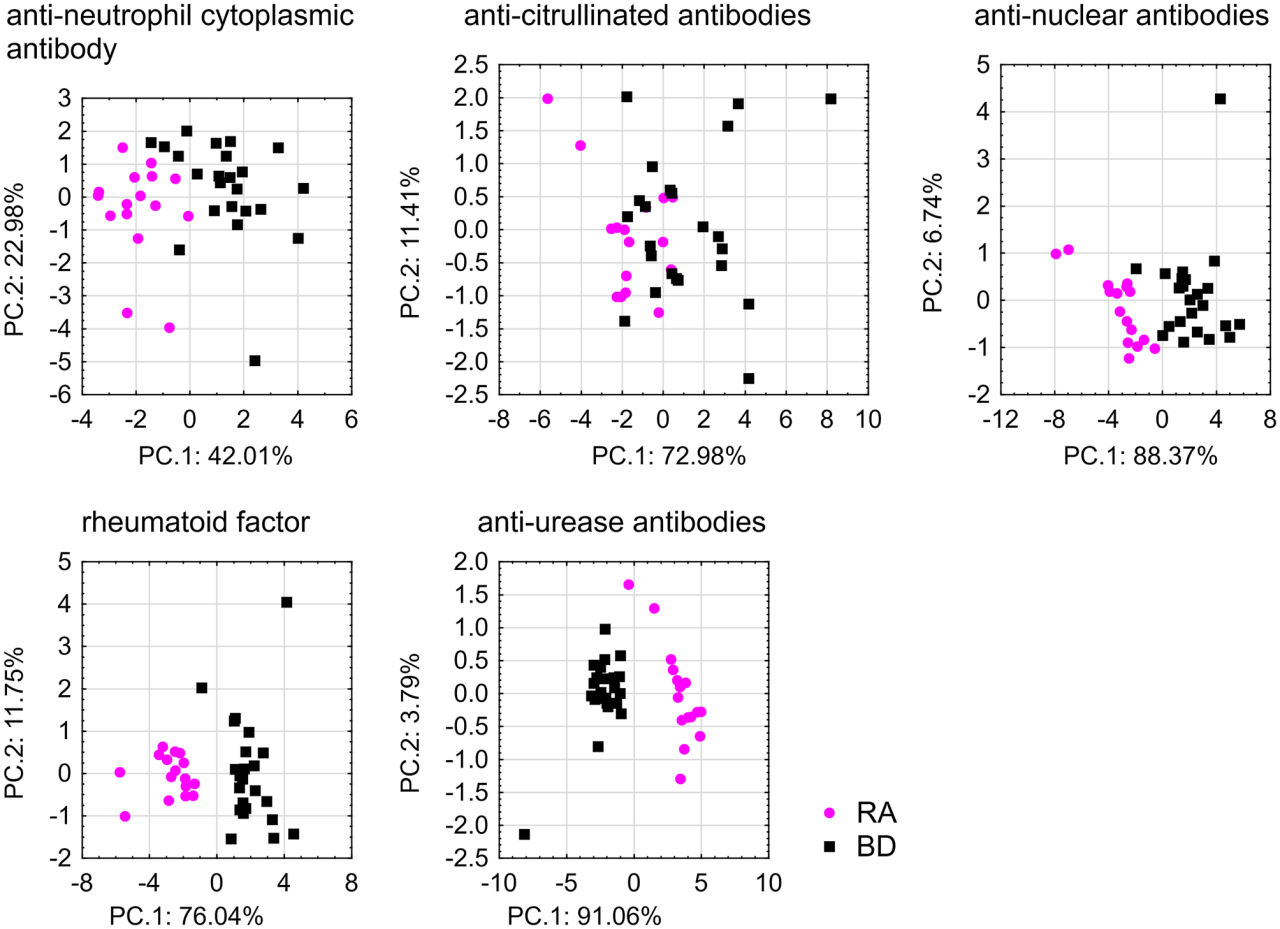
PCA scatter plot displaying the separation of rheumatic arthritis patients and healthy blood donors.

Subsequently, it was examined whether the selected predictors characteristic for their corresponded antibodies could be used to distinguish between patients and healthy donors. PCA analysis was used for differentiation of these spectral data into two categories, RA patients versus healthy blood donors. The PCA loadings and scatter plots display the spectral features characteristic of individual markers and samples for the first two components (Fig. 3). The wave numbers characteristic for anti-Ure antibodies and RF were the best at differentiating between the patient and healthy donor groups.

The above experiments investigated the correlation between examined antibodies and the wave number in the IR spectra of the tested sera. In the next step, it was examined whether wave numbers in the spectra exist that can distinguish patients from healthy donors in a specific and sensitive way, and whether the most strongly differentiating wave numbers correlate with any of the tested antibodies. The list of best predictors for distinguishing RA patients, calculated based on chi2 analysis and the Gini index, was similar to the list of predictors obtained for anti-Ure antibodies: 1103–1107, 1118–1120, 852, 1682, and 2982 cm^−1^. Additionally, there was a band at 1079 cm^−1^ (Table 2).

**Table 2:** Major band position elevated in rheumatoid arthritis patients along with the assignment of the bands.

ATR band positions wave number cm^–1^	Assignment	Reference value
1103–1107	Polysaccharide, RNA, ribose *v* (C=O),	1101, 1110^23,24^
1118–1120	RNA, *v* (C=O) ribose rings	1120^24–26^
1682	*v*_*as*_(C_2_=O) vibration respectively in RNA, amide I	1690, 1689–1678^24,27^
1079	DNA, RNA *v*_*s*_ (PO_2_^−^) _*sym*_. or phospholipid *v*_*s*_ (PO_2_^−^)	1080^23,24,26,28,29^
852	Oscillation of the P–O bond	855^30^
2982	C–H_*asym*_*.* > CH_2_ in fatty acids	2977^23,26,31^

**Figure 4:**
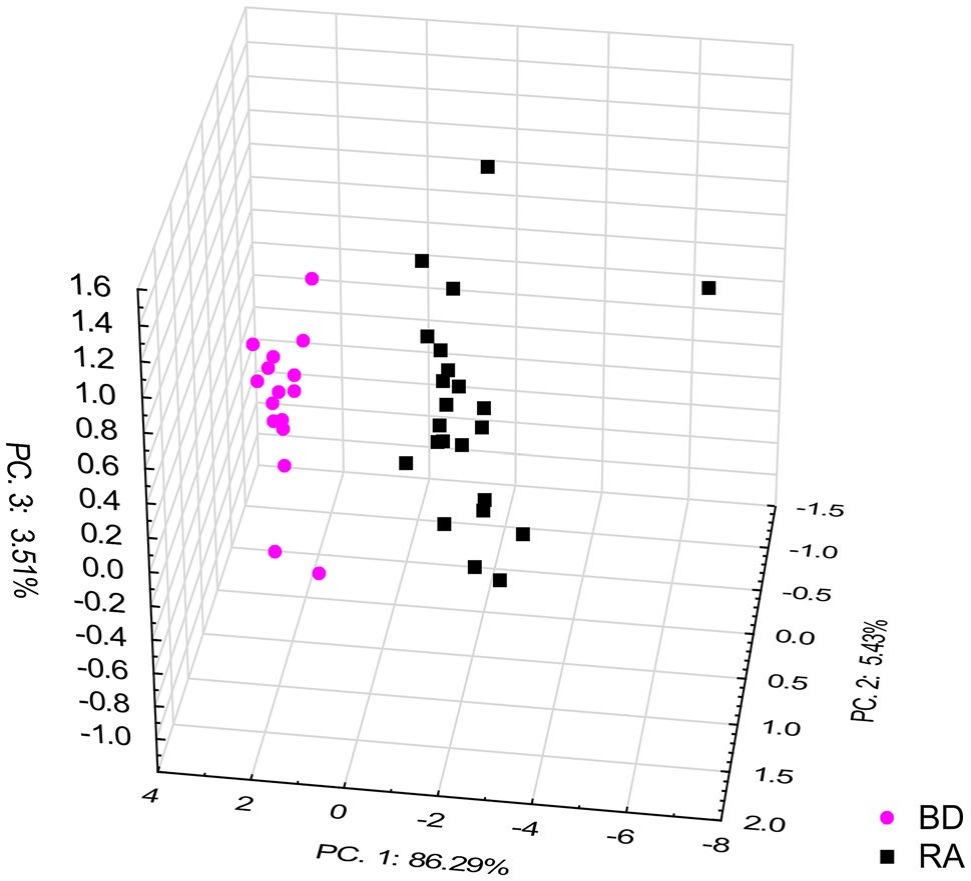
The PCA 3D scatter plot displaying the separation of rheumatoid arthritis patients and healthy blood donors based on wave numbers of 1120, 1105, 852, 2982, 1079, and 1682 cm^−1^.

**Figure 5:**
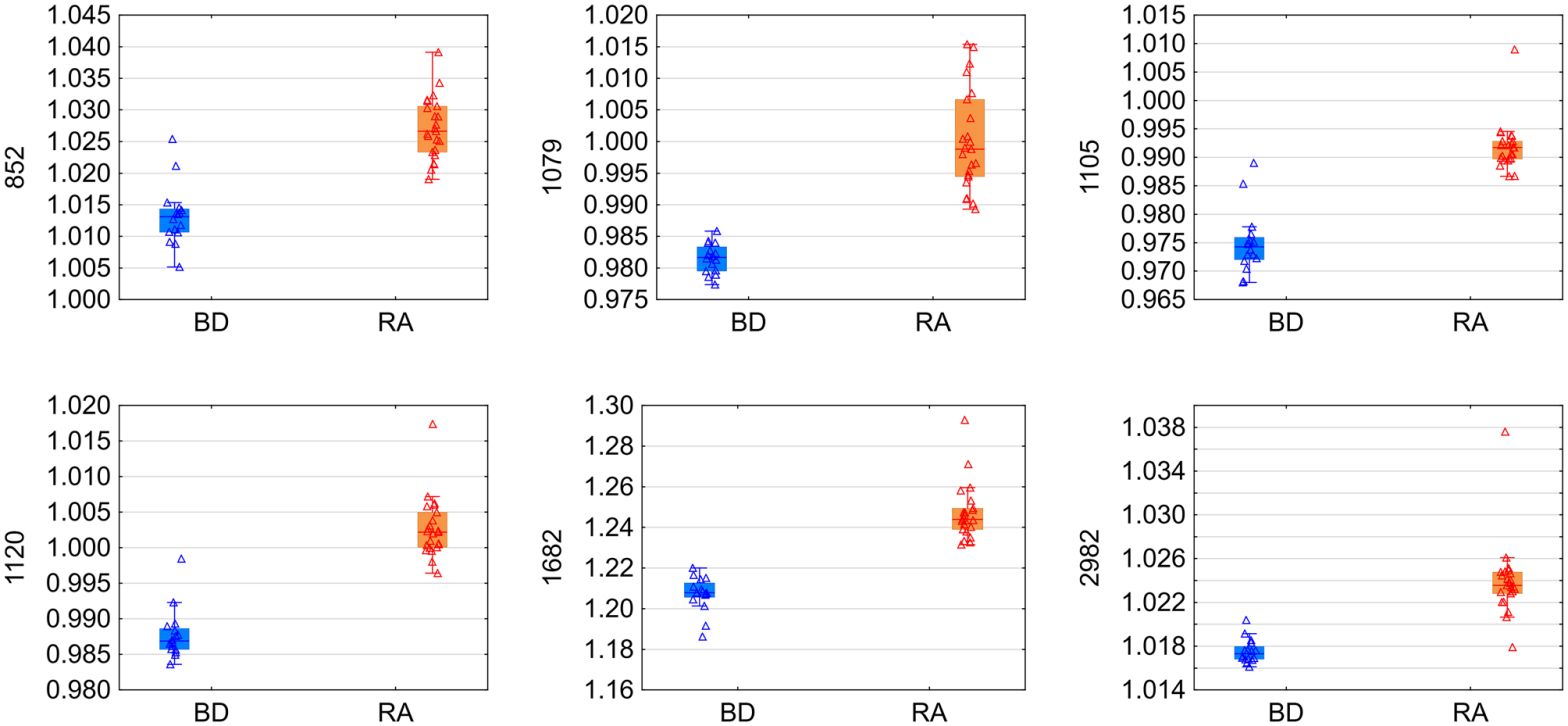
Longitudinal study of the intensity of the sera bands in rheumatoid arthritis patients (orange) compared with the healthy blood donors (blue). The intensities of all of the bands were significantly different between patients and healthy volunteers.

Reduction of these variables to those that were characteristic for the abovementioned regions (Table 2), made it possible to select statistically significant IR features as potential biomarkers of RA: 1120, 1105, 852, 2982, 1079, and 1682 cm^−1^, for inclusion in PCA. This spectral pattern correlation is illustrated in a three-dimensional (3D) scatter plot in Fig. 4. Taken together, all band intensities in patient samples were clearly distinguishable from those in the control group and were significantly different to those in healthy blood donors (*p* < 0.05) (Fig. 5).

These findings indicated the possibility that these spectral features (1120, 1105, 852, 2982, 1079, and 1682 cm^−1^) may be useful RA biomarkers in the future.

Moreover, using a DA model, the spectral markers were examined. Among the examined parameters, those with the greatest contribution to distinguishing RA patients from healthy blood donors corresponded to bands at 1105 and 1682 cm^−1^ (lowest partial Wilks’s lambda value). From the DA, discrimination of RA patients from healthy donors based on the mean values of these two parameters was statistically significant (Wilks' Lambda = 0.16935; F = 88.287; *p* < 0.0000) (Supplemental information 2. The calculated classification matrix showed that differentiating patients from healthy blood donors had a sensitivity and specificity of 100%.

## Discussion

Rheumatoid arthritis is one of the most common autoimmune diseases worldwide, and is characterized by strong heterogeneity in terms of disease manifestations, clinical outcomes, and the response to treatment^9,32–34^. Much research on RA focuses on finding new, more specific disease markers and techniques to facilitate basic research or rapid diagnosis. The continuous advances in technology aid the development of new research methods, as well as new applications for pre-existing research techniques. FTIR is one of the physical–chemical methods previously used in the field of chemistry that is now being increasingly applied in the fields of biology and medical sciences. Several studies have reported the application of FTIR to clinical chemistry analyses and disease diagnostics based on sera^19,22,35,36^, and there are already existing applications in various types of autoimmunological diseases and cancers^28,35,37–42^. However, so far there are few applications of this technique in RA. A recent report described the potential of this method to diagnose RA, but it did not report the selection of disease biomarkers^22^. In the study, the authors focused on the relationship between the IR sera spectra and antibodies present in these sera.

As a first step in this research, samples of the tested sera were characterized for the presence of ACPA, RF, ANA, ANCA, and anti-Ure antibodies. The autoantibodies selection criteria were occurrence and possible use in the diagnosis of RA. The results demonstrated that the RA patient sera contained mostly ACPA and RF, which is not surprising because RF and ACPA are important diagnostic markers of RA. The lower frequency of ANA and ANCA in the sera of patients with RA and their presence in a group of healthy donors indicated the reduced specificity of these autoantibodies in the context of RA, which is confirmed by the literature^3^. ACPA and RF were also present in the healthy donor sera, particularly ACPA, which was detected in 31% of the samples. The prevalence of RF and ACPA in the sera of healthy people is well-established, but some studies describe the lower percentage of ACPA-positive healthy individuals compared with RF-positive healthy individuals^6,11^. Despite the healthy donor sera being ACPA positive, the concentration of ACPA was significantly lower compared with that in RA patients, which was consistent with previous studies^6^.

A significant correlation was observed between most autoantibodies (with the exception of ANCA). The relationship between the occurrence of RF and ACPA is not surprising. Many previous studies have described the significant correlation between RF and ACPA, which may be associated with a seropositive diagnosis^6,32^. Data have also been reported on the correlation between the presence of RF, ACPA, and anti-carbamylated protein antibodies^32^, and the correlation between ANA and ACPA or RF has also been discussed^43,44^. The relationship between the various autoantibodies may be a result of disorders of the immune system.

In this study, anti-Ure antibodies were also analyzed. Studies of antibodies against urease in RA patient sera have primarily been described in the context of a bacterial role in RA development^45–47^, but recent studies have shown that anti-Ure antibodies may be a promising marker of RA, highlighting their high specificity for RA^15^. Increased levels of anti-Ure antibodies significantly correlated with the tested autoantibodies (with the exception of ANCA). These correlations may support the proposed importance of anti-Ure antibodies in the development of RA and the possibility of using anti-Ure antibodies as a marker of RA in the future.

As a result of the relationship between the presence of autoantibodies and anti-Ure antibodies, the discriminant ability of all markers, as well as each of them individually, was assessed. PCA indicated that the use of all analyzed markers did not allow for the correct identification of RA patients and healthy blood donors (Fig. 1a), whereas the removal of ANA and ANCA markers enabled the identification of RA patients and healthy blood donors (Fig. 1b). This information indicated that the detection of less specific markers such as ANA and ANCA may help in prognosis and the selection of appropriate treatment strategies, but will not facilitate diagnosis. The results showed that despite the fact that ANA and ANCA may be present in the sera of RA patients, the specificity was too low compared with other antibodies, as described previously^3^. Discriminant analysis confirmed these results. Among the examined parameters, the greatest contribution to distinguishing RA patients from healthy donors was found for anti-Ure antibodies (lowest partial Wilks’s lambda value) (Supplementary Material 1). The application of anti-Ure antibody analysis might be a potentially useful adjunct to current techniques for refining the classification of RA disease. However, these results describe findings from RA patients and healthy donors; there is no information about the presence of anti-Ure antibodies in other patients with autoimmune or rheumatic diseases.

Previous studies showed that sera from RA patients had a unique IR spectral pattern. Due to the large number of variables (4000–650 cm^−1^), a set of predictors was calculated for each of the analyzed autoantibodies. The chi2 test was used to select the wave numbers showing the greatest variability in relation to the studied grouping variable.

By applying ATR-FTIR spectroscopy to serum samples from RA patients and healthy blood donors, several characteristic spectral markers that correlated with the above-mentioned antibodies were identified (Fig. 2). The obtained results indicated a significant correlation between the selected predictors and autoantibodies. Correlations for the tested antibodies could be both positive and negative, which in the case of transformed IR spectra analysis did not necessarily translate directly into quantitative absorbance results. However, transformation of the IR spectra was necessary to reveal subtle differences between the spectra, which was not evident by assessing the absorbance levels alone.

Despite selection of the 1781 cm^−1^ and 1739 cm^−1^ wave numbers dependent on ANCA antibodies as the best predictors for classifying the sera samples using the chi2 test, Spearman’s rank correlation analysis showed no significant relationship between ANCA and these predictors. This result may be explained by the different algorithms and underlying principles of the two tests. Interestingly, both the 1781 cm^−1^ and 1739 cm^−1^ wave numbers are described as markers for lipids, C = O cholesteryl esters, and triglycerides^38^, and the third wave number 780 cm^−1^ is described as a marker for sugar phosphate vibrations^48^. There are also reports regarding the role of inflammation on lipid levels in RA. Earlier studies demonstrated a relationship between ANCA and lipids, and suggested a role for ANCA in the development of inflammation in ANCA-associated vasculitis patients. The suggested relationship between ANCA and wave numbers corresponding to lipids may be explained by the possibility that the presence of autoantibodies such as ANCA may be related to inflammation and may have an impact on disease manifestation^49^. The 1739 cm^−1^ band is also significantly correlated with the ACPA marker. The correlation between ACPA and the wave number characteristic for lipids and cholesterol suggests that it may also be associated with lipid lev els that may be dependent on inflammation levels. Furthermore, the characteristic bands for ACPA were 1340–1345 cm^−1^, associated with the amide III of proteins, 1197–1198 cm^−1^, associated with carbohydrates and creatinine, and 829 cm^−1^, associated with the aromatic C-H band^50^. The obtained results suggest that the presence of ACPA may affect the protein profile in sera samples, which can be explained by conformation changes and glycosylation of the autoantibodies.

ANA has been reported to mainly correlate with bands at 1620–1625 cm^−1^, a marker of the amide I β-sheet (IgG3, IgG2)^51^, 1200–1215 cm^−1^, markers of protein phosphorylation^52^, and 929 cm^−1^, the absorption band for carbohydrates^53^. The fact that ANA presence correlates with bands corresponding to proteins and antibodies may be explained by the unique chemical structure of the autoantibodies.

Characteristic bands for RF are mostly concentrated on protein bands: 1620–1630 cm^−1^ (amide I), 1510–1515 cm^−1^ (amide II), 938 cm^−1^ (phosphorylated proteins), and 1425/1180 cm^−1^ (amino acid bands)^51^. Correlations between bands within the protein window and autoantibodies may be a result of the structure of the antibodies and of immune complexes formed by the autoantibodies. Similarly, differences between bands within the carbohydrate window may be a result of glycosylation of proteins, including changes in glycosylation of antibodies that are characteristic of RA^8,12^.

Interestingly, anti-Ure antibodies, which seem to be important in the context of distinguishing RA patients from healthy blood donors, correlated with nucleic acid bonds and carbohydrates (1118–1120 cm^−1^, 1103–1107 cm^−1^, 2982 cm^−1^, and 852 cm^−1^)^24^. It is difficult to link the presence of anti-Ure antibodies with ribose and nucleic acids. Many studies have indicated increased levels of different types of RNA in the blood of RA patients, including microRNAs, long non-coding RNAs, and circular RNAs differentially expressed in RA, which are described as promising markers for RA diagnosis and treatment^54,55^. The increased absorbance of wave numbers characteristic of RNA in patient sera may not be caused by the presence of anti-Ure antibodies, but rather nucleic acid markers associated with the development of the disease may coexist with anti-Ure antibodies.

Considering data on the relationship between anti-Ure antibodies and the differences in the spectra of RA patients’ sera, it was examined whether anti-Ure antibody correlated wave numbers may be useful for differentiating patients from healthy donors.

Using the chi2 method and validation with the Gini index (most often used in the construction of classification trees**)**, we determined the best predictors for distinguishing RA patients from healthy donors. The 1118–1120, 1103–1107, 2982, and 852 cm^−1^ bands were also characteristic of anti-Ure antibodies. An additional predictor was the 1079 cm^−1^ band, which could be assigned to the symmetrical phosphodiester stretch of nucleic acids^28^ but this might also appear in the IgG spectrum. A positive band at 1682 cm^−1^ was assigned to an RNA and amide I^24^.

Reduction of the number of predictors to five characteristic spectral markers (1105, 1120, 1079, 1682, and 852 cm^−1^) identified in the sera of RA patients, improved the classification results, which seemed to be linked to the presence of RNA/DNA in serum. Discriminant analysis showed that two wave numbers sufficed to distinguish RA sera from healthy blood donor sera, namely, 1105 cm^−1^ and 1682 cm^−1^. In the literature, wave number 1105 cm^−1^ was used to develop the experimental model of *H. pylori* infection in guinea pigs^56^ and dengue virus in blood^57^. Both are cases of infectious diseases, which may indicate the association of this band with infection. Since inflammation is one of the symptoms of RA, and infectious agents are considered one of the etiological factors of the disease, it is possible that the 1105 cm^−1^ band is a marker of inflammation. The band at 1682 cm^−1^ is described in the literature as a marker of protein aggregation and conformation change. Most reports on this subject have been in the context of the diagnosis of neurodegenerative diseases, such as Parkinson's and Alzheimer’s disease, where the aggregation of proteins is important^58^. This biomarker was also used in studies on hypothyroidism^59^. These findings may indicate that both wave numbers 1105 cm^−1^ and 1682 cm^−1^ are effective in distinguishing RA patients from health blood donors, but they are probably not specific for RA and may instead be more general markers of homeostasis disorders. In an animal model reported by Titus et al., the wave number at 1292 cm^−1^ was shown to be a good biomarker of arthritis. This peak was identified as thymine, which resulted from the breakdown of thymidine in the sera of arthritis patients. Interestingly, in our work, this wave number was also significantly correlated with RA, as well as with RF and urease. However, it was less significantly correlated than the bands at^25^ 1105 and 1682 cm^−1^.

Our studies of RA using FTIR spectroscopy revealed differences in the biochemical profiles between patients and healthy blood donors, as shown by various changes in the carbohydrate and phosphate window, which mainly corresponded to nucleic acid characteristic wave numbers. The main changes observed in sera between the groups were in several functional groups of nucleic acids. Particularly, when studying the IR spectra of sera, it was found that the frequency of oscillations in the structure of DNA and RNA played a key role in discrimination, which could be explained by the presence of non-coding nucleic acids in RA and various types of autoimmunological diseases^55,60–62^. Circulating DNA and glycemic profiling have also proven to be critical molecular markers in several cancers^30,36^. Significant differences in the lipid, protein, and carbohydrate constituents of sera have been observed, but these differences are not sufficient to distinguish RA patients from healthy blood donors using mathematical methods similar to those used for autoantibodies.

The FTIR spectra of biological systems are complex due to the overlapping absorption of multiple components. A limitation of our studies was the relatively small number of sera samples. Moreover, in studies using only RA patients’ sera, it was not known whether the observed changes in the spectra were characteristic of RA or other diseases involving autoimmune disorders. A larger number of patients with more diverse rheumatic and autoimmune diseases will need to be explored to establish the value of the spectral markers identified in the present study. However, the relative simplicity and convenience in handling serum samples, and the potential for higher throughput, compared with other methods currently being applied to RA diagnosis, makes this a worthwhile endeavor.

The results obtained by ATR-FTIR showed that this technique may be useful in the study of RA patients' sera samples. The correlations identified shed new light onto the study of autoantibodies and the potential applications of ATR-FTIR in autoimmune disease studies.

## Materials and methods

### Sera samples

Healthy blood donors (16 females, mean age 45.5 ± 5) were from the Swietokrzyskie Blood Center in Kielce and patients with diagnosed RA (19 females and four males, mean age 63 ± 13 years) were under the care of the Swietokrzyskie Rheumatology Center in Konskie. Informed consent was obtained for experimentation. The sera were aliquoted and stored at − 20 °C for further analysis. Samples were thawed directly before use in experiments. The ACPA concentration and the presence and titer of RF, were measured as previously described^63^. The levels of the sera reaction to urease mimicking peptides were as described previously^15^. The median serum antibody level (for ACPA, RF, and anti-Ure) was calculated using STATISTICA (StatSoft, Round Rock, TX, USA). Samples were collected with the approval of the Ethics Committee of the Regional Chamber of Physicians in Kielce No. 30/2019-VII, 17.10.2019, and all methods were performed in accordance with the relevant guidelines and regulations.

### ANA and ANCA detection

The presence of ANA was measured by an enzyme immunoassay method using the ANA-Screening Enzyme Immunoassay Microplate Test (BioSystem, Barcelona, Spain) according to the manufacturer’s instructions. The presence of ANCA was detected using Anti-Neutrophil Cytoplasmic Antibodies Indirect Fluorescence Human Neutrophils (BioSystem) according to the manufacturer’s instructions. The levels of C reactive protein (CRP), the erythrocyte sedimentation rate (ESR), and the levels of RF, ACPA, ANA, ANCA, and anti-Ure antibodies, as well as information about treatment, are shown in Table 1.

### ATR-FTIR spectroscopy and multivariate analysis

The IR spectra of sera samples were as described previously^22^. The chi2 statistical test was used to check the region of the IR spectra that correlated with the examined feature. The results were then analyzed by a pairwise principal component analysis (PCA) for exploration of the existence of patterns in the multivariate IR data set, and discrimination of spectroscopic changes between the RA and healthy blood donor groups. For analysis based on the screen plot, sufficient principal components were selected to explain at least 75% of the variance. This qualitative analysis enabled collection of information about the latent structure of the spectral matrix, and it was an important source of knowledge to evaluate the suitability of posterior discriminant methods.

Linear discriminant analysis was applied for two different purposes, to discriminate RA from healthy blood donor samples based on: (1) molecular markers, and (2) spectral samples. For the selection of variables retained for the model, the Wilks' lambda method was applied. In addition, the Mann–Whitney test was conducted to verify the significance of the selected spectral markers. The value of *p* < 0.05 was considered statistically significant. Spearman’s rank correlation was used for the calculation of continuous variables and Kendall’s tau correlation was used for the calculation of nominal variables. STATISTICA (StatSoft) was used for all statistical analyses.

## Supplementary Information

Supplementary Information.
