# Supplementary material for: Correlations between autoantibodies and the ATR-FTIR spectra of sera from rheumatoid arthritis patients
Source: Sci Rep. 2021 Sep 9;11:17886. doi: 10.1038/s41598-021-96848-w (PMC8429563; doi:10.1038/s41598-021-96848-w)
Supplement: Supplementary file 1 — Supplementary Information. [file 41598_2021_96848_MOESM1_ESM.docx]

**Supplementary materials**

S 1. Results of discriminant function analysis

| N=39 | Wilks' Lambda = 0,36941; F =19,915; p< ,0000 | | | |
| --- | --- | --- | --- | --- |
|  | \| Wilks' Lambda \| \| --- \| | \| Partial Lambda \| \| --- \| | \| F \| \| --- \| | \| p \| \| --- \| |
| \| Anti-Ure \| \| --- \| | 0,764584 | 0,483149 | 37,44144 | 0,000001 |
| \| RF \| \| --- \| | 0,416542 | 0,886845 | 4,46577 | 0,041783 |
| \| ACPA \| \| --- \| | 0,388297 | 0,951354 | 1,78969 | 0,189592 |

S 2. Results of discriminant function analysis

| N=39 | Wilks' Lambda = 0,16935; F = 88,287; p< ,0000 | | | |
| --- | --- | --- | --- | --- |
|  | \| Wilks' Lambda \| \| --- \| | \| Partial Lambda \| \| --- \| | \| F \| \| --- \| | \| p \| \| --- \| |
| 1105 | 0,257077 | 0,658765 | 18,64769 | 0,000118 |
| 1682 | 0,243191 | 0,696380 | 15,69594 | 0,000337 |

S 3. Scree plot of for three clinical markers PC1 and PC2 accounted for 88.37% of the total variance.
